# Vascular endothelial growth factor-C (VEGF-C) expression in human colorectal cancer tissues

**DOI:** 10.1054/bjoc.2000.1396

**Published:** 2000-09-04

**Authors:** K Akagi, Y Ikeda, M Miyazaki, T Abe, J Kinoshita, Y Maehara, K Sugimachi

**Affiliations:** Department of Surgery and Science, Graduate School of Medical Sciences, Kyushu University, 3-1-1 Maidashi, Higashi-ku, Fukuoka 812-8582, Japan

**Keywords:** VEGF-C, lymphatic involvement, lymph nodes metastasis, colorectal carcinoma

## Abstract

Vascular endothelial growth factor-C (VEGF-C) functions specifically to induce lymphangiogenesis. We examined the relationship between expression of VEGF-C and clinicopathological features in patients with colorectal cancer. The expression of VEGF-C in the 99 primary tumours and 18 metastatic lymph nodes from colorectal cancer patients was examined immunohistochemically. To verify VEGF-C mRNA expression, reverse transcriptase-polymerase chain reaction (RT-PCR) was carried out. The expression of VEGF-C correlated with lymphatic involvement, lymph nodes metastasis, and depth of invasion. On the other hand, correlations were nil with regard to gender of the patients, histologic type, venous involvement, and liver metastasis. The expression of VEGF-C in metastatic lymph nodes was fairly consistent with this expression in the primary tumour. Survival time was shorter for VEGF-C positive groups than for VEGF-C negative ones, but with no statistically significant difference. RT-PCR findings revealed that the expression of VEGF-C mRNA correlated mostly with that of VEGF-C protein expression. VEGF-C may play an important role in lymphatic spread of colorectal cancer. © 2000 Cancer Research Campaign


*British Journal of Cancer* (2000) **83**(7), 887–891
© 2000 Cancer Research Campaign
doi: 10.1054/ bjoc.2000.1396, available online at http://www.idealibrary.com on IDEAL®

### Vascular endothelial growth factor-C (VEGF-C) expression in human colorectal cancer tissues

**K Akagi, Y Ikeda, M Miyazaki, T Abe, J Kinoshita, Y Maehara and K Sugimachi**

Department of Surgery and Science, Graduate School of Medical Sciences, Kyushu University, 3-1-1 Maidashi, Higashi-ku, Fukuoka 812-8582, Japan

**Summary** Vascular endothelial growth factor-C (VEGF-C) functions specifically to induce lymphangiogenesis. We examined the relationship between expression of VEGF-C and clinicopathological features in patients with colorectal cancer. The expression of VEGF-C in the 99 primary tumours and 18 metastatic lymph nodes from colorectal cancer patients was examined immunohistochemically. To verify VEGF-C mRNA expression, reverse transcriptase-polymerase chain reaction (RT-PCR) was carried out. The expression of VEGF-C correlated with lymphatic involvement, lymph nodes metastasis, and depth of invasion. On the other hand, correlations were nil with regard to gender of the patients, histologic type, venous involvement, and liver metastasis. The expression of VEGF-C in metastatic lymph nodes was fairly consistent with this expression in the primary tumour. Survival time was shorter for VEGF-C positive groups than for VEGF-C negative ones, but with no statistically significant difference. RT-PCR findings revealed that the expression of VEGF-C mRNA correlated mostly with that of VEGF-C protein expression. VEGF-C may play an important role in lymphatic spread of colorectal cancer. © 2000 Cancer Research Campaign

**Keywords:** VEGF-C; lymphatic involvement; lymph nodes metastasis; colorectal carcinoma

Colorectal cancer is a common cause of death throughout the world, and lymph nodes metastasis is an important prognostic factor (Dukes and Bussey, 1958; Newland et al, 1981; Chapuis et al, 1985; Fielding et al, 1986). The 5-year survival rate in patients with colorectal cancer with lymph nodes metastasis is worse than those without lymph nodes metastasis (Hermanek, 1995).

VEGF-C was initially identified to be a factor stimulating tyrosine kinase receptor Flt4 (VEGFR-3), which was purified from PC-3 prostatic adenocarcinoma cells (Joukov et al, 1996). The VEGF-C gene is localized on chromosome 4q34 (Paavonen et al, 1996) and has a high degree of homology to VEGF (Joukov et al, 1997). The open reading frame of VEGF-C c-DNA encodes a protein of 419 amino acid residues, with a predicted molecular mass of 46.9kDa (Joukov et al, 1997). Its mRNA is 2.4 and 2.0 kb (Joukov et al, 1997), which is expressed in human adult tissues, including heart, placenta, muscle, ovary, and small intestine (Joukov et al, 1996). The expression of VEGF-C was also detected in several types of malignant tumours (Salven et al, 1998). VEGF-C is a ligand for VEGFR-3, which is predominantly expressed in the endothelium of lymphatic vessels (Kaipainen et al, 1995; Kukk et al, 1996), and VEGF-C is considered to be both a specific marker for lymphatic endothelial cells and a specific factor for lymphangiogenesis.

To date, only a few clinicopathological studies on VEGF-C expression in malignant tumours have been reported (Ohta et al, 1999; Tsurusaki et al, 1999; Valtola et al, 1999; Yonemura et al, 1999).

*Received 10 February 2000*
*Revised 31 May 2000*
*Accepted 8 June 2000*

*Correspondence to:* Kazunari Akagi

To better understand the mechanism of lymph nodes metastasis in cases of colorectal cancer, we examined the relationship between expression of VEGF-C and clinicopathological features, using immunohistochemical techniques and RT-PCR. Our data show a strong correlation between VEGF-C expressions and lymph nodes metastasis.

### MATERIALS AND METHODS

### Tissue specimens

We studied 99 Japanese colorectal cancer patients who had been surgically treated in Department of Surgery and Science, Graduate School of Medical Sciences, Kyushu University, Fukuoka, Japan between 1991 and 1995. Age of the patients ranged from 15 to 89 years (mean 63 years), and 57 were men and 42 were women. For immunohistochemistry, tissue specimens were obtained from all 99 primary colorectal cancers, and lymph nodes specimens with metastatic tumours were selected from 18 cases.

For RNA extraction, 17 pairs of resected primary colorectal carcinoma tissue and corresponding normal tissues were immediately placed in liquid nitrogen. No patient had been given neo-adjuvant or adjuvant therapy. All pathological and histological classifications of the tumours were based on the TNM classification (Sobin and Wittekind, 1997).

### Immunohistochemistry

The surgical specimens were fixed in 10% formalin solution and embedded in paraffin. Histological slices of 3 μm were prepared, then were deparaffined in xylene, and dehydrated with ethanol. Endogenous peroxidase was blocked with 0.3% $H_2O_2$ in methanol for 20 min at room temperature. After washing with phosphate-buffered saline (PBS), non-specific binding was blocked by treating with 10% normal rabbit serum for 40 min at room temperature. Anti-VEGF-C antibody (goat, polyclonal. Santa Cruz, California) was applied on the histological sections at a dilution of 1:50 and incubated in a moist chamber overnight at 4°C. After washing the specimens with PBS, the slides were incubated in biotinylated rabbit anti-goat antibody for 20 minutes at room temperature. After three washes in the PBS, sections were incubated in streptavidin-peroxidase reagent for 5 minutes at room temperature. VEGF-C antigen was developed using diaminobenzidine (Merck, Germany) and 2 mM hydrogen peroxide in 0.05% PBS for 3 min at room temperature. For counterstaining, we used Mayer's Hematoxylin. A negative control was achieved by adding a blocking peptide (Santa Cruz, California) to the primary antibody. No staining was observed in the section.

### Evaluation of immunoreactivity

Results of the immunohistochemical staining were evaluated by a pathologist blinded to all clinical data. To evaluate the protein expression, the results were graded as follows: (+) over 10% of the neoplastic cells were stained, (–) completely negative. In this study, (–) and (±) were classified as negative, (+) was classified as positive.

### Microvessel staining and counting

For microvessel staining, we randomly selected 20 samples from the VEGF-C positive and negative groups, respectively. The method of microvessel staining and counting were done as described (Tomisaki et al, 1996). Briefly, intratumoral microvessels were highlighted by immunostaining with a mouse Mab against CD34 (Novocastra, UK) in a 1:100 dilution and incubated overnight at 4°C. Any single brown-stained cell or cluster of endothelial cells clearly separated from adjacent vessels, tumour cells and other connective tissue elements were considered to be a microvessel. The stained sections were screened at ×40 magnification to identify regions of highest vascular density within the tumour. Vessels were counted in the 3 regions of highest vascular density at ×200 magnification (0.739 m m$^2$ per field) The microvessel numbers were the mean number of vessels in these areas.

### RT-PCR amplification

Total RNA was isolated using ISOGEN (Nippon Gene, Inc, Tokyo, Japan) RNA extraction kit and reverse-transcribed using murine leukaemia virus reverse transcriptase. Quantity and quality of mRNA from all samples were certified by RT-PCR amplification of the GAPDH gene. Amplifications of VEGF-C transcripts (763-bp) were done using the Perkin-Elmer GeneAmp PCR system 9700 (Norwalk, CT, USA) and oligodeoxynucleotide primers (forward; 5′-ACCTGCCCCACCAATTACA-3′, reverse; 5′-GCCTCTTGTAAAGACTGGTT-3′). These primers for VEGF-C were designed from previously published sequences (Wartiovaara et al, 1998). Thermal conditions of the system were as follows; one cycle at 95°C for 5 min; 35 cycles at 95°C for 1 min, 52°C for 1 min, 72°C for 1.5 min; one cycle at 72°C for 7 min. The amplified DNA fragment was electrophoresed on 1.2% agarose gels containing ethidium bromide with a DNA molecular weight marker for comparison.

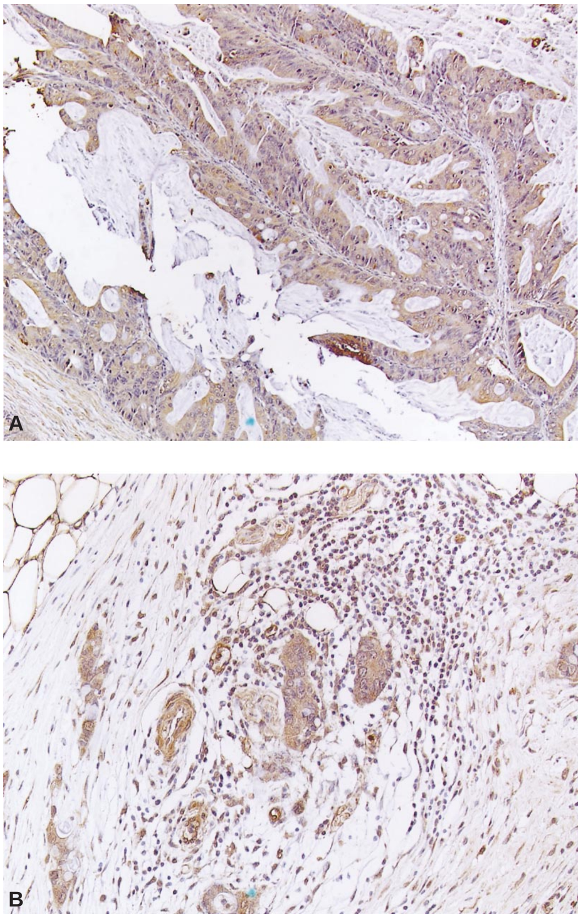

**Figure 1** (**A**) Positive immunoreactivity for VEGF-C (primary tumour). Magnification ×100. (**B**) Positive immunoreactivity for VEGF-C (metastatic lymph nodes). Magnification ×200

### Statistical analysis

The BMDP Statistical Package program (BMDP, Los Angeles, CA) for the main frame computer (4381; IBM, Armonk, NY) was used for all analyses. Associations between the variables were tested using Student's *t*-test or Fisher's exact probability test. The BMDP LR program was used for multivariate adjustments for all covariates simultaneously, with a backward stepwise logistic regression analysis.

### RESULTS

### Immunohistochemistry

In specimens of normal colorectal mucosa, no VEGF-C protein was stained. Among the 99 examined tumours, 55 showed VEGF-C protein expression in the cytoplasm (Figure 1A). However, no expression was observed in the nucleus area. In 44 of 99 colorectal cancer specimens, VEGF-C was not expressed. The relationship between VEGF-C expression and clinicopathological features is given in Table 1. The positive expression of VEGF-C was significantly higher ($P$ = 0.0032) in tumours with lymphatic involvement (50.9%) than in those without lymphatic involvement (29.5%). The positive expression of VEGF-C was significantly higher ($P$ = 0.0025) in tumours with lymph nodes metastasis (60.0%)

 *© 2000 Cancer Research Campaign*

**Table 1** Expression of VEGF-C and clinicopathological features

| | *n* | VEGF-C expression Positive | VEGF-C expression Negative | *P* value |
|---|---|---|---|---|
| Mean age (yrs) ± SD | | 61.8 ± 12.9 | 64.0 ± 11.1 | NS |
| Gender | | | | NS |
| Men | 57 | 28 | 29 | |
| Women | 42 | 27 | 15 | |
| Tumour diameter (mm) ± SD | | 49.4 ± 17.8 | 44.4 ± 24.1 | NS |
| Histologic type | | | | NS |
| Well | 45 | 27 | 18 | |
| Moderately | 47 | 25 | 22 | |
| Poorly | 7 | 3 | 4 | |
| Depth of invasion | | | | 0.0359 |
| Tis,T1,T2 | 18 | 6 | 12 | |
| T3,T4 | 81 | 49 | 32 | |
| Lymphatic involvement | | | | 0.0032 |
| Negative | 58 | 27 | 31 | |
| Positive | 41 | 28 | 13 | |
| Venous involvement | | | | NS |
| Negative | 54 | 29 | 25 | |
| Positive | 45 | 26 | 19 | |
| Lymph nodes metastasis | | | | 0.0025 |
| Negative | 53 | 22 | 31 | |
| Positive | 46 | 33 | 13 | |
| Liver metastasis | | | | NS |
| Negative | 89 | 47 | 42 | |
| Positive | 10 | 8 | 2 | |
| Microvessel numbers | | 64.9 ± 15.1 | 67.9 ± 14.6 | NS |

NS, no significant difference; SD, standard deviation

**Table 2** Multivariate analyses with respect to lymphatic involvement and lymph nodes metastasis

| Variable | Relative risk | *P* value |
|---|---|---|
| Lymphatic involvement | | |
| Venous involvement | 9.62 | 0.0001 |
| Lymph nodes metastasis | 3.33 | 0.0187 |
| VEGF-C | 3.24 | 0.028 |
| Lymph nodes metastasis | | |
| Lymphatic involvement | 4.86 | 0.0002 |
| VEGF-C | 3.26 | 0.006 |

**Table 3** Relationship between VEGF-C expression in primary tumours and that in metastatic lymph nodes

| | Primary tumours Positive (*n* = 12) | Primary tumours Negative (*n* = 6) | *P* value |
|---|---|---|---|
| Metastatic lymph nodes + | 11 | 2 | 0.0217 |
| Metastatic lymph nodes – | 1 | 4 | |

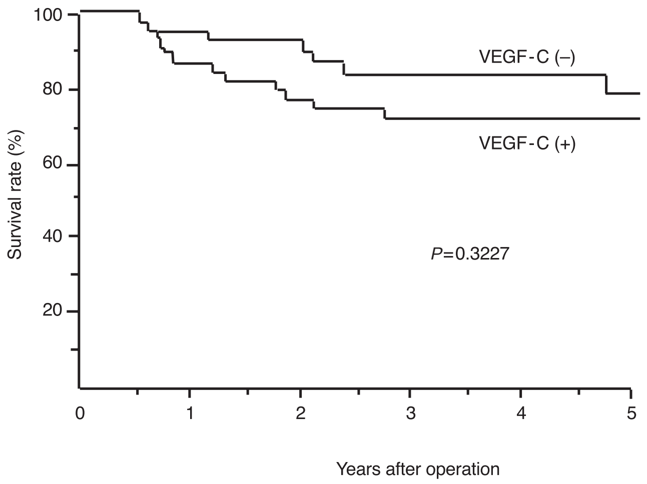

**Figure 2** Survival curves for patients with colorectal cancer, based on the expression of VEGF-C. There were 55 patients with positive expression of VEGF-C (dark line) and 44 patients with negative expression of VEGF-C (light line), with no statistical differences in survival between the two groups than in those without lymph nodes metastasis (29.5%). The rate of positive expression of VEGF-C also increased with the depth of tumour invasion ($P = 0.0359$). Correlation among gender of the patients, histologic type, venous involvement, liver metastasis and microvessel numbers was nil.

Multivariate analyses showed VEGF-C expression to be an independent factor influencing both lymphatic involvement and lymph nodes metastasis (Table 2).

The expression of VEGF-C in metastatic tumours in lymph nodes was compared with that in primary tumours. Among the 18 cases examined (47 metastatic lymph nodes), 13 (40 metastatic lymph nodes) showed a positive expression of VEGF-C protein. VEGF-C was expressed in the cytoplasm of metastatic cancer cells, and was the same as expression in the primary tumours (Figure 1B). The relationship between VEGF-C expression in primary tumours and that in metastatic lymph nodes is given in Table 3. The expression of VEGF-C in metastatic lymph nodes was fairly consistent with expression in the primary tumour.

Five-year survival for the patient population was 73 percent, and survival rates were poorer for patients in the VEGF-C positive

*© 2000 Cancer Research Campaign* 

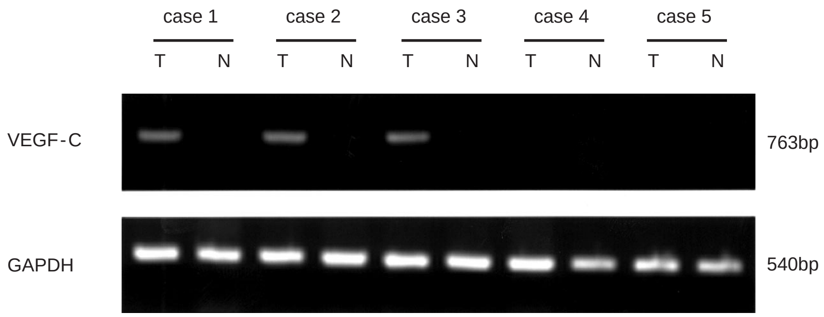

**Figure 3** Expression of VEGF-C mRNA in representative cases of VEGF-C protein positive group (cases 1–3) and negative one (cases 4, 5). T, tumour tissue; N, normal tissue. GAPDH is used as an internal control. Expression was measured using RT-PCR

group than those in the negative group, but the difference was not statistically significant (Figure 2).

### mRNA expression

17 pairs of primary colorectal carcinoma tissue and corresponding normal tissue were examined to verify VEGF-C mRNA expression. RT-PCR was done using primers of VEGF-C and those of GAPDH as a internal control. Figure 3 shows examples of VEGF-C mRNA expression. Seven of 10 VEGF-C protein positive cases showed VEGF-C mRNA expression, on the other hand, only one of seven VEGF-C protein negative cases showed VEGF-C mRNA expression. The expression of VEGF-C mRNA was mostly correlated with that of VEGF-C protein expression.

### DISCUSSION

In this study, VEGF-C was highly expressed in the primary tumour of patients with both lymph nodes metastasis and lymphatic involvement, and VEGF-C expression increased in the metastatic tumour of lymph nodes, as compared with findings in the primary tumour. On the other hand; the expression of VEGF-C was not related to hematogenous metastasis, such as liver metastasis or venous involvement. These findings suggest that VEGF-C was functionally associated with lymphatic involvement and lymph nodes metastasis.

There have been few studies on the relationship between VEGF-C and other malignant diseases (Ohta et al, 1999; Tsurusaki et al, 1999; Valtola et al, 1999; Yonemura et al, 1999), and we find no documentation of the relationship between colorectal cancer and the immunohistochemical expression of VEGF-C. We obtained evidence that VEGF-C is expressed in the cytoplasm of colorectal cancer cells, findings consistent with reported data (Yonemura et al, 1999). In cases of gastric cancer, the expression of VEGF-C, determined using RT-PCR and immunohistochemistry, was strongly associated with lymph nodes metastasis and patients with a high expression of VEGF-C protein had a significantly poorer prognosis than those with low VEGF-C expression (Yonemura et al, 1999). In cases of human malignant mesothelioma tumours, a strong association between microlymphatic vessel density and VEGF-C mRNA expression was also observed, however, expression of VEGF-C showed no correlation with lymph nodes metastasis and prognosis (Ohta et al, 1999). In cases of human prostatic carcinoma, the expression of VEGF-C, examined using the situ hybridization, showed that VEGF-C mRNA was significantly stronger in the lymph node-positive group than in the lymph node-negative group (Tsurusaki et al, 1999). Our findings that VEGF-C is associated with lymphangiogenesis in colorectal cancer are consistent with these previous results.

The VEGF family is mediated by three known tyrosine kinase receptors, VEGFR-1, 2, and 3. VEGF is a ligand for VEGFR-1 (Flt-1) and 2 (KDR/Flk-1) (Ferrara and Davis-Smyth, 1997). VEGFR-2 leads to proliferation and migration of endothelial cells, inducing angiogenesis. On the other hand, VEGFR-1 cannot induce angiogenesis when stimulated with VEGF (Landgren et al, 1998). VEGFR-1 (Flt-1) and 2 (KDR/Flk-1) are expressed on vascular endothelial cells (Ferrara and Davis-Smyth, 1997). VEGFR-3 differs from VEGFR-1 and VEGFR-2 in that it is mainly expressed in the endothelium of lymphatic vessels (Kukk et al, 1996), which means that it may be specific marker for lymphatic endothelial cells. VEGF-C is a ligand for VEGFR-3 (Joukov et al, 1996), and is thought to be a specific factor inducing lymphangiogenesis. Overexpression of VEGF-C in the skin of transgenic mice resulted in lymphatic endothelial proliferation and vessel enlargement (Jeltsch et al, 1997). Furthermore, VEGF-C was found to have a specific function in lymphangiogenesis. Therefore, the increased expression of VEGF-C in colorectal cancer with lymph nodes metastasis may be due to the specific function of VEGF-C in lymphangiogenesis.

As VEGF-C is also a ligand for VEGFR-2, it may have angiogenic effects. In the present study, CD34 immunostaining was done to measure numbers of microvessels. However, we observed no significant difference in these numbers between VEGF-C positive and negative groups, which indicates that VEGF-C has little or no angiogenic effect, at least, in colorectal cancer.

We also tried to measure lymphatic vessel numbers using VEGFR-3 immunohistochemistry because it had been considered to be the only specific marker for lymphatic endothelial cells. However, VEGFR-3 expression was recognized in the endothelium of blood vessels as well as in that of lymphatic vessels (those vessels had previously been identified by H.E. staining). Thus, it seems unlikely that VEGFR-3 is a specific marker for lymphatic vessels. This finding parallels data of other investigators (Partanen et al, 1999). A more sensitive way to detect lymphatic vessels has to be designed.

We also noted the correlation between expression of VEGF-C and depth of the tumour. As a tumour penetrates, it is more likely to make contact with lymphatic vessels in the submucosal layer. Therefore, whether the increased expression of VEGF-C with tumour depth is due to the malignant potential of tumour invasion to the deep layer or to the increased potential of lymphangiogenesis secondary to tumour growth has to be determined.

 *© 2000 Cancer Research Campaign*

In the multivariate analyses, the potential of venous involvement was closely related to that of lymphatic involvement. Molecular mechanisms differ between venous and lymphatic involvement of cancer cells. For example, VEGF is related to venous involvement (Kitadai et al, 1998) and VEGF-C to lymphatic involvement (Yonemura et al, 1999). However, these tumour advances were commonly and concomitantly noted in clinical colorectal cancers (Tsuchiya et al, 1995).

Survival rates were poorer in VEGF-C positive groups than in VEGF-C negative ones, but the difference was not statistically significant. VEGF-C, which is closely related to lymph nodes metastasis, may influence survival rates. However, hematogenous metastasis, such as liver metastasis, is also an important factor for survival rates of colorectal cancer patients (Scheele et al, 1990; Sugihara et al, 1993). Therefore, in contrast to another study (Yonemura et al, 1999), VEGF-C may not statistically influence survival rates in colorectal cancer patients.

As VEGF-C expression is associated with lymph nodes metastasis in colorectal cancer patients, this expression may aid in detecting lymphatic spread, such as lymphatic involvement and lymph nodes metastasis.

### ACKNOWLEDGEMENTS

We are grateful to M. Ohara for providing helpful comments on the manuscript and J. Tsuchihashi for technical assistance. This study was supported by grants from the Ministry of Education, Science, Sports and Culture of Japan.

### REFERENCES

Chapuis PH, Dent OF, Fisher R, Newland RC, Pheils MT, Smyth E and Colquhoun K (1985) A multivariate analysis of clinical and pathological variables in prognosis after resection of large bowel cancer. *Br J Surg* **72**: 698–702

Dukes CE and Bussey HJR (1958) The spread of rectal cancer and its effect on prognosis. *Br J Cancer* **12**: 309–320

Ferrara N and Davis-Smyth T (1997) The biology of vascular endothelial growth factor. *Endocr Rev* **18**: 4–25

Fielding LP, Phillips RK, Fry JS and Hittinger R (1986) Prediction of outcome after curative resection for large bowel cancer. *Lancet* **2**: 904–907

Hermanek P (1995) pTNM and residual tumor classifications: problems of assessment and prognostic significance. *World J Surg* **19**: 184–190

Jeltsch M, Kaipainen A, Joukov V, Meng X, Lakso M, Rauvala H, Swartz M, Fukumura D, Jain RK and Alitalo K (1997) Hyperplasia of lymphatic vessels in VEGF-C transgenic mice. *Science* **276**: 1423–1425

Joukov V, Pajusola K, Kaipainen A, Chilov D, Lahtinen I, Kukk E, Saksela O, Kalkkinen N and Alitalo K (1996) A novel vascular endothelial growth factor, VEGF-C, is a ligand for the Flt4 (VEGFR-3) and KDR (VEGFR-2) receptor tyrosine kinases. *EMBO J* **15**: 1751

Joukov V, Kaipainen A, Jeltsch M, Pajusola K, Olofsson B, Kumar V, Eriksson U and Alitalo K (1997) Vascular endothelial growth factors VEGF-B and VEGF-C. *J Cell Physiol* **173**: 211–215

Kaipainen A, Korhonen J, Mustonen T, van Hinsbergh VW, Fang GH, Dumont D, Breitman M and Alitalo K (1995) Expression of the fms-like tyrosine kinase 4 gene becomes restricted to lymphatic endothelium during development. *Proc Natl Acad Sci USA* **92**: 3566–3570

Kitadai Y, Haruma K, Tokutomi T, Tanaka S, Sumii K, Carvalho M, Kuwabara M, Yoshida K, Hirai T, Kajiyama G and Tahara E (1998) Significance of vessel count and vascular endothelial growth factor in human esophageal carcinomas. *Clinical Cancer Research* **4**: 2195–2200

Kukk e, Lymboussaki A, Taira S, Kaipainen A, Jeltsch M, Joukov V and Alitalo K (1996) VEGF-C receptor binding and pattern of expression with VEGFR-3 suggests a role in lymphatic vascular development. *Development* **122**: 3829–3837

Landgren E, Schiller P, Cao Y and Claesson-Welsh L (1998) Placenta growth factor stimulates MAP kinase and mitogenicity but not phospholipase C-gamma and migration of endothelial cells expressing Flt 1. *Oncogene* **16**: 359–367

Newland RC, Chapuis PH, Pheils MT and MacPherson JG (1981) The relationship of survival to staging and grading of colorectal carcinoma: a prospective study of 503 cases. *Cancer* **47**: 1424–1429

Ohta Y, Shridha V, Bright RK, Kalemkerian GP, Du W, Carbone M, Watanabe Y and Pass HI (1999) VEGF and VEGF type C play an important role in angiogenesis and lymphangiogenesis in human malignant mesothelioma tumours. *Br J Cancer* **81 (1)**: 54–61

Paavonen K, Horelli-Kuitunen N, Chilov D, Kukk E, Pennanen S, Kallioniemi OP, Pajusola K, Olofsson B, Eriksson U, Joukov V, Palotie A and Alitalo K (1996) Novel human vascular endothelial growth factor genes VEGF-B and VEGF-C localize to chromosomes 11q13 and 4q34, respectively. *Circulation* **93**: 1079–1082

Partanen TA, Alitalo K and Miettinen M (1999) Lack of lymphatic vascular specificity of vascular endothelial gorwth factor receptor 3 in 185 vascular tumors. *Cancer* **86**: 2406–2412

Salven P, Lymboussaki A, Heikkila P, Jaaskela-Saari H, Enholm B, Aase K, von Euler G, Eriksson U, Alitalo K and Joensuu H (1998) Vascular endothelial growth factors VEGF-B and VEGF-C are expressed in human tumors. *Am J Pathol* **153**: 103–108

Scheele J, Stangl R and Altendorf-Hofmann A (1990) Hepatic metastases from colorectal carcinoma: impact of surgical resection on the natural history. *Br J Surg* **77**: 1241–1246

Sobin LH and Wittekind C (1997) TNM classification of malignant tumours. *International Union Against Cancer* Fifth edition: 66–73

Sugihara K, Hojo K, Moriya Y, Yamasaki S, Kosuge T and Takayama T (1993) Pattern of recurrence after hepatic resection for colorectal metastases. *Br J Surg* **80**: 1032–1035

Tomisaki S, Ohno S, Ichiyoshi Y, Kuwano H, Maehara Y and Sugimachi K (1996) Microvessel quantification and its possible relation with liver metastasis in colorectal cancer. *Cancer* **77**: 1722–1728

Tsuchiya A, Ando Y, Kikuchi Y, Kanazawa M, Sato H and Abe R (1995) Venous invasion as a prognostic factor in colorectal cancer. *Surg Today* **25**: 950–953

Tsurusaki T, Kanda S, Sakai H, Kanetake H, Saito Y, Alitalo K and Koji T (1999) Vascular endothelial growth factor-C expression in human prostatic carcinoma and its relationship to lymph node metastasis. *Br J Cancer* **80**: 309–313

Valtola R, Salven P, Heikkila P, Taipale J, Joensuu H, Rehn M, Pihlajaniemi T, Weich H, de Waal R and Alitalo K (1999) VEGFR-3 and its ligand VEGF-C are associated with angiogenesis in breast cancer. *Am J Pathol* **154**: 1381–1390

Wartiovaara U, Salven P, Mikkola H, Lassila R, Kaukonen J, Joukov V, Orpana A, Ristimaki A, Heikinheimo M, Joensuu H, Alitalo K and Palotie A (1998) Peripheral blood platelets express VEGF-C and VEGF which are released during platelet activation. *Thromb Haemost* **80**: 171–175

Yonemura Y, Endo Y, Fujita H, Fushida S, Ninomiya I, Bandou E, Taniguchi K, Miwa K, Ohoyama S, Sugiyama K and Sasaki T (1999) Role of vascular endothelial growth factor C expression in the development of lymph node metastasis in gastric cancer. *Clin Cancer Res* **5**: 1823–1829

© 2000 Cancer Research Campaign 
